# Healthy ageing: Herpes zoster infection and the role of the adjuvanted recombinant zoster vaccine

**DOI:** 10.1093/eurpub/ckac131.013

**Published:** 2022-10-25

**Authors:** D Curran, TM Doherty, N Lecrenier, T Breuer

**Affiliations:** 1 Value Evidence, GlaxoSmithKline, Wavre, Belgium; 2 Medical/Vaccines, GlaxoSmithKline, Wavre, Belgium; 3 Medical Governance & Bioethics, GlaxoSmithKline, Wavre, Belgium

## Abstract

**Background:**

Increases in life expectancy over the last 50 years has been matched by an increase in the burden of diseases (e.g. herpes zoster (HZ)) in adults ≥50 years of age (YOA). Without intervention, around 30% of individuals can expect to develop HZ in their lifetime, which would impact their daily activities and healthy ageing.

**Methods:**

We conducted a narrative review on published literature on the impact of developing HZ on healthy ageing and the ability of vaccination to prevent the burden of disease due to HZ. Specifically, we describe HZ impact on quality of life (QoL), and impact of the adjuvanted recombinant zoster vaccine (RZV) on reducing the burden of HZ in adults ≥50 YOA.

**Results:**

In adults ≥50 YOA with HZ, 65.1% and 15.8% reported severe pain and worst imaginable pain, respectively. Pain persisted for up to 90 days (defined as post-herpetic neuralgia) in 10-20% of HZ patients, and occasionally for years after initial symptoms. Pain due to HZ impacted all domains of QoL (psychological, physical and social). Evidence suggested that RZV reduced HZ burden of illness and burden of interference on daily activities by > 90%. Reports also suggested that RZV retained vaccine efficacy of > 90% in all frailty subgroups, who typically respond poorly to other vaccinations. Long-term follow-up data reported vaccine efficacy against HZ of 84.1% (95% confidence interval, 64.4% - 94.0%), 8 years post-vaccination. Modelling studies demonstrated that vaccination resulted in reduced hospitalization and other healthcare visits related to HZ.

**Conclusions:**

Vaccination with RZV can protect older adults from HZ, thus maintaining QoL and promoting active and healthy ageing.

**Key messages:**

• There is significant burden of disease due to HZ among adults ≥50 YOA due to ageing and immunosenescence.

• Vaccination can reduce burden of disease among the elderly and frail individuals and maintain QoL.

